# Effect of Heat Treatment on the Grain Boundary Character Distribution and Bending Properties of Fine-Grained Phosphorus Bronze

**DOI:** 10.3390/ma18091941

**Published:** 2025-04-24

**Authors:** Zhongping Chen, Yang Yang, Huafen Lou, Hu Wang

**Affiliations:** 1 School of Material Science and Engineering, Central South University, Changsha 410083, China; czp2987@126.com; 2 Chinalco Research Institute of Science and Technology Co., Ltd., Beijing 102209, China; hu_wang625@chinalco.com.cn

**Keywords:** grain boundary engineering, electron backscatter diffraction, special boundaries, fine grain, phosphorus bronze

## Abstract

Grain boundary engineering (GBE) has been widely used to modify grain boundary (GB) networks to improve GB-related properties in polycrystalline materials. With the development of miniaturized and lightweight terminal connectors comes a greater demand for phosphorus bronze. A fine grain size and excellent GB characteristics are the keys to synergistically enhancing mechanical strength and bending workability. In this study, the effects of the annealing temperature on the grain boundary character distribution (GBCD) optimization and the bending properties of phosphorus bronze were studied by means of electron backscatter diffraction and a 90° bending test. The results show that the deformed microstructure of the as-received material recrystallizes upon annealing at 400 °C for 1 h. The average grain size is 1.6 μm, and a large number of special boundaries (SBs) are present, accounting for 71.5% of all GBs. Further, the incoherent Σ3, Σ9, and Σ27 boundaries are the most abundant, effectively disrupting the network connectivity of random high-angle grain boundaries. The grain size gradually increases with the annealing temperature increase. The fractions of the Σ9 and Σ27 boundaries gradually decrease. Although the proportion of SBs further increases at higher temperatures, most SBs at these temperatures are coherent Σ3 boundaries that do not contribute to the direct optimization of GBCD. Moreover, in the absence of a significant difference in tensile strength, the GBCD-optimized fine-grained sample demonstrates smooth surfaces without orange peel effects when bent at 90° with R/t = 0 in the bad way. This improvement is attributed to the uniform deformation of fine grains and special boundaries, which enhances the bending workability of the GBCD-optimized fine-grained strips.

## 1. Introduction

As is well known, the number, type, and distribution of grain boundaries (GBs) in polycrystalline materials significantly affect the material properties. Numerous studies have shown that many behaviors of polycrystalline materials, such as intergranular precipitation [1,2,3,4], corrosion [5,6], oxidation [7], fracture [8,9], and ductility [10,11,12], are closely related to the grain boundary structure. In recent decades, people have attempted to improve the properties of materials by controlling and optimizing the grain boundary character distribution (GBCD) through grain boundary engineering (GBE) [13,14,15,16,17]. GBE is based on specific thermomechanical processing (TMP) aimed at increasing the fraction of special boundaries (SBs) in the low Σ coincidence site lattice (CSL), where SBs are defined by their close-fitting interfacial geometry, which disrupts the connectivity of the random high-angle grain boundary (RHAGB) network (Σ > 29) [17,18,19,20]. Low ΣCSL boundaries with 3 ≤ Σ ≤ 29 were defined as low-energy SBs, where Σ represents the reciprocal of the CSL density. Compared with RHAGBs (Σ > 29), low ΣCSL (3 ≤ Σ ≤ 29) boundaries exert a stronger inhibitory effect on corrosion, fracture, and other factors and are believed to possess unique inherent properties, such as a low interfacial energy [18]. In contrast, because of their lower structural order, larger free volume, and higher interfacial energy, RHAGBs (Σ > 29) often act as the nuclei for crack initiation and propagation.

GBE thermomechanical processing (TMP) is the main strategy for realizing the GBCD optimization of materials with a medium-to-low stacking fault energy (SFE) [14,15]. TMP can be used as a preliminary step to create a fine-grained structure, which can then be further optimized by GBE to adjust the GBCD. This sequential application can lead to a material with an even more refined and optimized microstructure. Studies have shown that materials suitable for GBCD are usually limited to those that can generate a large number of Σ3 and Σ3-related boundaries [17]. In practice, most successful GBE applications that result in property improvements have n values of 1 ≤ n ≤ 3, where the Σ3 boundaries include both coherent (Σ3_c_, immobile) and incoherent (Σ3_ic_, mobile) twins. Therefore, controlling and optimizing the grain boundary structure of polycrystalline materials to improve GB-related properties are effective methods.

Phosphorus bronze is a commonly used elastic copper alloy with excellent mechanical properties attributed to the presence of Sn and P and cold work hardening. Further, it can be easily processed into various complex shapes for use in elastic components. In addition to its excellent elastic properties, phosphorus bronze is corrosion- and wear-resistant and nonmagnetic. It is currently the most widely used elastic material in copper-based elastic alloy materials. Bending is a basic formation method used to manufacture terminal connectors; hence, the manufacturability and the reliability of terminal connectors depend on the bending properties of copper alloy strips. With the miniaturization of electronic components, the components used for terminal connectors have also been miniaturized, consequently decreasing the bending radius of the components and making the site of bending more prone to phenomena, such as orange peeling and cracking. Phosphorus bronze is widely used in terminal connectors, which require high bendability and strength to satisfy the growing requirements of electronic components [21].

Li et al. [22] systematically investigated the effects of annealing, aging, and cold deformation on the microstructural evolution and bending properties of Cu-Cr-Zr alloy strips. The study demonstrated that the refined microstructure of Cu-Cr-Zr alloys significantly influences bending properties, exhibiting superior bendability. Gao et al. [23] examined the effects of low-temperature annealing processes on the microstructure and bending properties of C19400 copper alloy strips. Their findings revealed that grain refinement significantly improves the bendability of the C19400 alloy, thereby meeting the miniaturization demands required for high-end connectors. Oda et al. [24] investigated the fraction of low-Σ grain boundaries (Σ3, Σ5, Σ7) in aluminum alloys and found that these low-order boundaries exhibit superior energy stability, whereas higher-Σ grain boundaries tend to initiate cracks accompanied by significant stress concentration phenomena. The results demonstrate that both grain size and grain boundary character exert significant influences on the bending properties of the alloy. Grain refinement and boundary type optimization can effectively enhance the bending properties of alloys. The key lies in precise microstructural control, where thermomechanical processing enables simultaneous refinement and boundary type optimization. Phosphorus bronze is a face-centered cubic metal with a low SFE; thus, its microstructure is expected to be optimized through GBE TMP and result in a fine grain structure, a higher fraction of low ΣCSL grain boundaries, high strength, and improved strip bending properties. However, the specific details about the mechanism of the microstructural evolution of phosphorus bronze and its bending properties remain largely unknown.

Therefore, after achieving below 2 μm fine grains through TMP, 20% cold rolling reduction is used in this study. The microstructure and GBCD at different annealing temperatures are assessed through electron backscatter diffraction (EBSD). Furthermore, the effect of GBCD optimization on the bending properties of phosphorus bronze strips is explored.

## 2. Experimental Materials and Methods

The as-received material was a phosphorus bronze strip. Initially, the alloy was annealed to achieve a recrystallized microstructure with grain sizes below 2 μm, followed by a 20% cold rolling reduction. Its chemical composition is shown in Table 1. The as-received material was annealed at temperatures ranging from 300 °C to 700 °C for 1 h and was then air-cooled. The samples annealed at 400 °C and 600 °C were cold rolled to achieve comparable mechanical properties, with tensile strengths of approximately 620 MPa. These samples were designated as the GBCD-optimized fine-grained sample and the unoptimized coarse-grained sample, respectively. The samples were collected along the longitudinal section of the strip (RD–ND direction). The samples were mechanically ground using 180-, 800-, and 2500-grit diamond sandpaper, followed by polishing with a polishing cloth. Subsequently, they were electrolytically polished for 10 s at 25 V and 0.8 mA in a 2:2:5 (by volume) phosphoric acid–absolute ethanol–deionized water mixed solution.

The Vickers hardness tests were performed at room temperature using a TH730 digital display Vickers hardness tester. The tests were conducted with a selected load of 0.1 kgf and a dwell time of 10 s. The Vickers hardness value for each sample was determined by averaging three valid measurements after excluding the highest and lowest values from five test points. Tensile tests were conducted at room temperature using an Instron 5967-30KN universal testing machine. To ensure experimental reproducibility and stability, three parallel specimens were tested for each sample under a constant strain rate of 1 × 10^−3^ s^−1^.

The orientation data were collected using a FEI Apreo C field-emission scanning electron microscope (Hillsboro, OR, USA) interfaced to an EDAX EBSD system. The data were processed using OIM Analysis 8.0 software. All the EBSD scans were conducted at a working voltage of 20 kV. On the basis of the grain size of the analyzed samples, the step size for EBSD scanning ranged from 0.001 μm to 0.5 μm. The CSL model and the Brandon criterion [25] were used to classify the Σ boundaries. The accuracy of the misorientation measurements obtained by EBSD was 0.5°. In this study, the CSL boundaries with 3 ≤ Σ ≤ 29 were defined as low-energy SBs [26,27], while GBs with Σ > 29 were classified as RHAGBs. To ensure the accuracy of grain size and Σ statistics, five different regions of each sample were measured during data collection. The fraction of each SB type was defined as the ratio of the length of the SBs to the total length of all GBs. To obtain the statistical significance, the length fractions of the GBs were determined in different scanning areas. The average grain size was calculated from the equivalent grain diameter. Based on the distribution of the image quality (IQ) map, the strain distribution was qualitatively analyzed. The IQ map measures the clarity of the Kikuchi mode, and the higher the lattice strain, the lower the clarity. However, because the IQ map depends on the grain orientation and the slight inhomogeneity of the surface, the combination of the IQ and inverse pole figure (IPF) maps is necessary.

To elucidate the evolution of GBs during the phosphorus bronze annealing, in situ heating EBSD observations were conducted on samples annealed at 400 °C for 15 min and 30 min in this study. The region of interest was first located at room temperature. The sample was heated, and after reaching the set temperature, EBSD scanning was initiated at different holding time intervals.

To evaluate the bending workability of phosphorus bronze, the bending property is defined herein as the ratio of the bending radius (R) to the sheet thickness (t) expressed as (R/t) and calculated accordingly. The bending workability was evaluated according to the ASTM B820 standard [28]. A 90° bending test was conducted by means of an HSL-BT-90 bending tester (Cheshire, UK). The samples were 15 mm in width and 20 mm long and were bent at 90° in a bad way (BW). Observations were conducted using a Keyence VHX-5000 ultra-depth microscope (Osaka, Japan). The bending properties of the samples were classified according to five ranks: Rank 1 (smooth), Rank 2 (small orange peel), Rank 3 (heavy orange peel), Rank 4 (shallow cracks), and Rank 5 (deep cracks). Additionally, metallographic (OM) and EBSD microstructural characterizations were performed on the cross-sections and the surfaces of the tensile side of the bending samples.

## 3. Results and Discussion

### 3.1. Effect of Annealing Temperature on Microstructure and the GBCD

**Figure 1:**
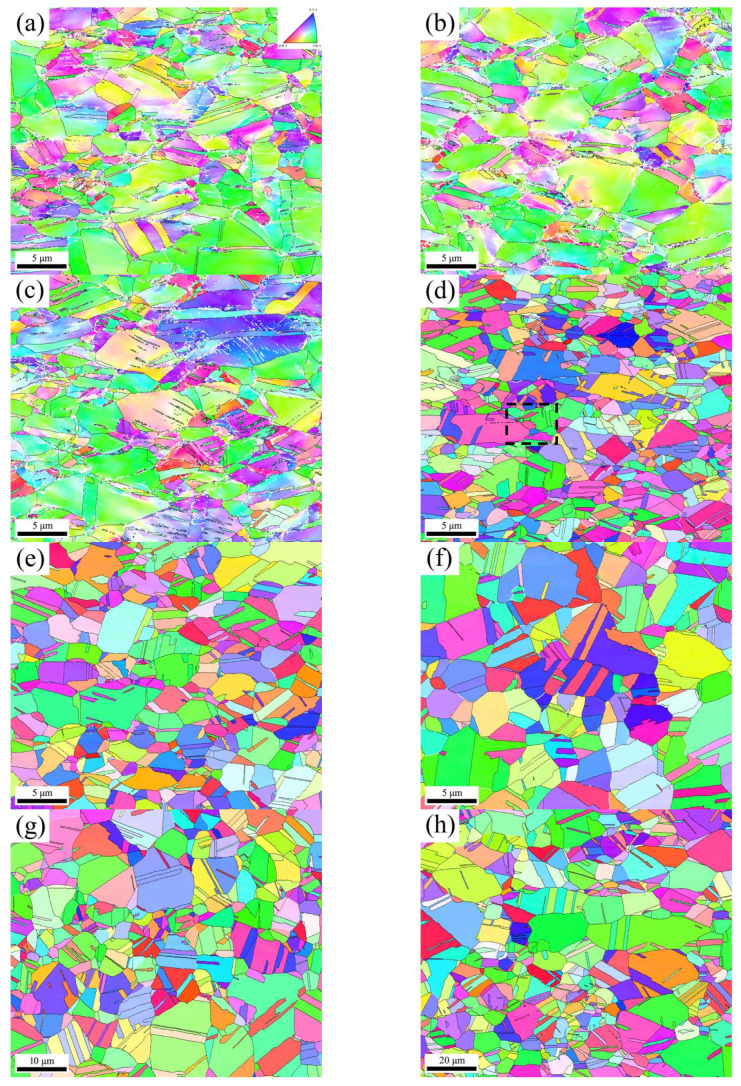

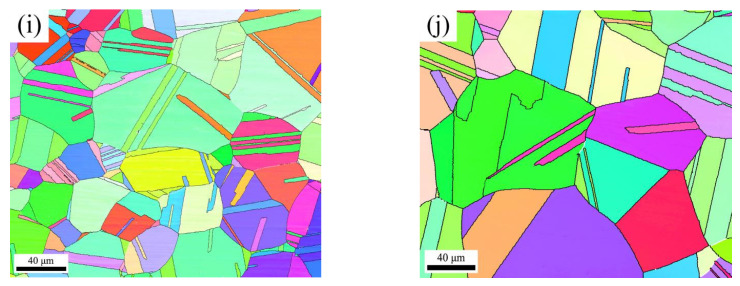
Inverse pole figure (IPF) maps of the as-received sample (**a**) and the phosphorus bronze samples annealed at 300 °C (**b**), 350 °C (**c**), 400 °C (**d**), 450 °C (**e**), 500 °C (**f**), 550 °C (**g**), 600 °C (**h**), 650 °C (**i**), and 700 °C (**j**) for 1 h. The color codes for the grains are represented by the standard triangle in (**a**), with black lines indicating high-angle grain boundaries (HAGBs) and white lines indicating low-angle grain boundaries (LAGBs).

Figure 1 shows an inverse pole figure (IPF) maps of the as-received sample and the phosphorus bronze samples annealed at different temperatures for 1 h. The grain orientations are represented by colors in the standard triangle shown in Figure 1a. The high-angle grain boundaries (HAGBs, θ > 15°) and the low-angle grain boundaries (LAGBs, 2° < θ < 15°) are represented by the black and white lines, respectively. Figure 1a shows the deformed microstructure of the as-received sample, with a gradient change in the grain orientation and a high density of LAGBs confirming the presence of a large number of dislocations in the as-received sample. Moreover, some twin-like fragments are also present. The relatively small deformation indicates that they are mainly annealing twins (approximately hundreds of nanometers to several microns) within the previous annealing and deformation twins (approximately several nanometers to dozens of nanometers) formed during deformation. Due to the thinness of the deformation twins, which are on the nanometer scale, the selected EBSD scanning step size (0.1 μm) is relatively large, preventing a full visualization of the deformation twins and causing a majority of them to be overlooked.

**Figure 2:**
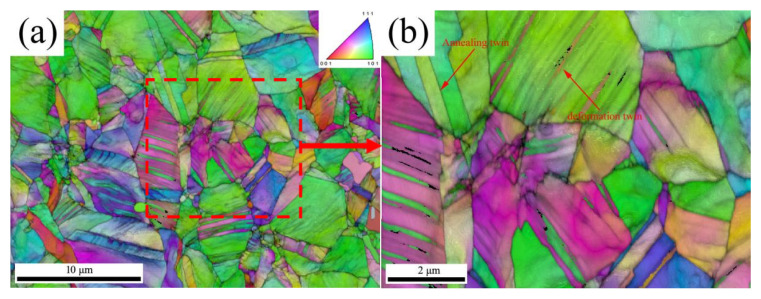
IPF and IQ combined map of the as-received sample under transmission Kikuchi diffraction (TKD) mode during EBSD. (**a**) as-received sample (**b**) as-received sample magnified region.

**Figure 3:**
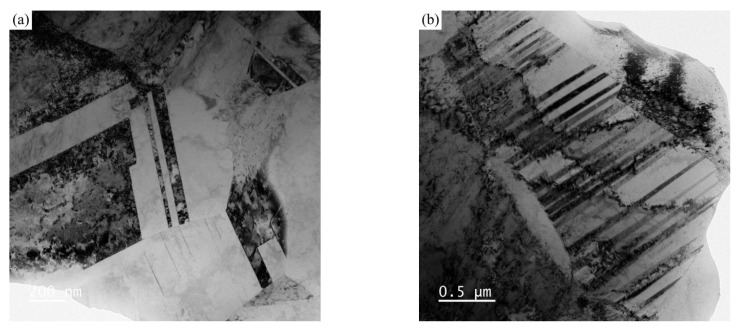
TEM micrographs of the deformed microstructure in the as-received sample. (**a**) dislocation-rich regions and dislocation-free regions (**b**) deformation twin with high dislocation density region.

A scanning step size as low as 10 nm was achieved by utilizing the transmission Kikuchi diffraction (TKD) mode during EBSD (Figure 2). Figure 2a shows many deformation twins within the local grains in addition to the annealing twins. These deformation twins are arranged in parallel (Figure 2b). On the basis of their morphology, these can be inferred as deformation twins [29,30,31]. Figure 3 shows TEM micrographs of the deformed microstructure in the as-received sample. From the TEM micrograph in Figure 3a, it can be observed that there are numerous dislocation-rich regions and dislocation-free regions in the annealing twins of the as-received sample. Meanwhile, the TEM image in Figure 3b reveals the presence of a large number of parallel thin twin lamellae within a single grain, with a width of approximately several tens of nanometers. Therefore, the deformed microstructure of the as-received sample mainly results from the heterogeneity caused by the dislocation slip and the deformation twins.

The fundamental principle of strain-induced boundary migration (SIBM) is the directional movement of grain boundaries driven by strain energy. During plastic deformation, dislocation accumulation within grains results in localized energy increases and an inhomogeneous dislocation density distribution, thereby generating the driving force for grain boundary migration. Grain boundaries are inclined to migrate toward regions with higher stored strain energy in order to minimize the total system energy. During this process, local bulging occurs at grain boundaries on the high-strain side, acting as nuclei for recrystallization. These bulged regions progressively propagate into high-strain zones, consuming the deformed matrix and forming new strain-free grains [32,33].

Table 2 illustrates the variation in grain size and Σ boundary fractions (%) across five sampled regions (a–e) in 400 °C-annealed samples. The findings demonstrated that there was little intra-sample variation in the Σ values and grain sizes. The results are similar to Randle‘s statistical results [34].

**Figure 4:**
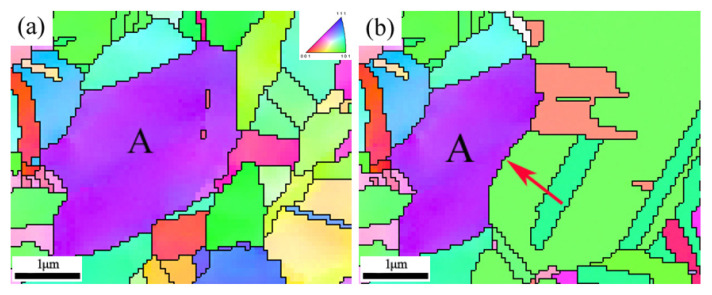
In situ recrystallization IPF maps of the annealed samples at 400 °C for 15 min (**a**) and 30 min (**b**).

**Figure 5:**
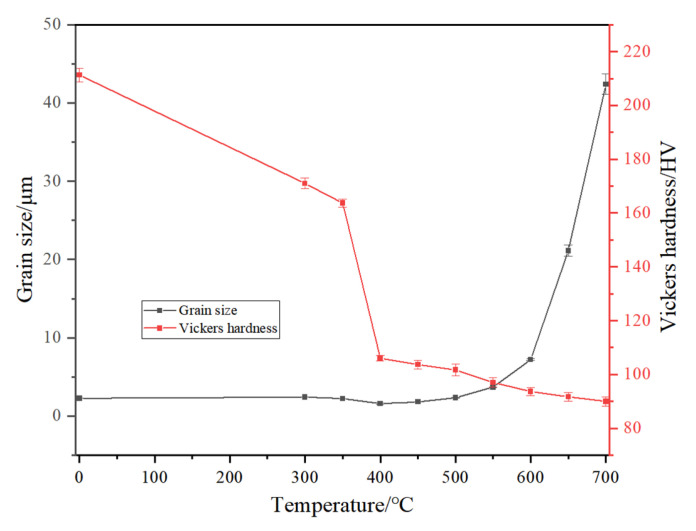
Vickers hardness and grain sizes (including twins) of the as-received sample and annealed samples at 300~700 °C for 1 h.

For the samples annealed at 300 °C and 350 °C, the microstructures shown in Figure 1b,c are essentially the same as those of the as-received sample, with many dislocations and deformation twins. These results indicate that within this temperature range, the annealed samples undergo only recovery, and there is no interface migration or SIBM. The IPF map in Figure 1d and the results in Table 3 show that increasing the temperature to 400 °C results in a significant reduction in the density of LAGBs, decreasing to 4.5%. Figure 4 clearly depicts the SIBM process. As shown in Figure 4, local bulging occurs at the grain boundary on the high-strain side of deformed grain A (purple region), serving as a nucleation site for recrystallization. The primary recrystallization grains are engulfing the deformed grains in region A (indicated by the purple area), with the red arrows showing the direction of migration of the new grains into the deformed matrix. Subsequently, many annealing twins form at the recrystallization front [9,30,35,36], and the number of annealing twins significantly increases. The number of LAGBs is greatly reduced with the release of the stored energy. Very few LAGBs remain after annealing at 400 °C for 1 h. Upon completion of recrystallization, the grain orientation is changed, resulting in a random distribution. The Vickers hardness test results reveal that the hardness value is half that of the as-received sample (Figure 5). Compared to that of the as-received sample, the average grain size in the treated sample is essentially unchanged, with a value of approximately 1.6 μm, including the twins. At 450 °C (Figure 1e), the grain size is not significantly different (approximately 1.8 μm), and the number of LAGBs continues to decrease. The grain orientation is essentially the same as that at 400 °C. The moderate amount of strain (20% reduction) is sufficient for generating a relatively large number of recrystallization nucleation sites, inducing the SIBM. Ultimately, the SIBM occurs via the HAGB migration along high-residual stress regions [37,38]. After these migrating grains collide with each other, the GBCD remains essentially unchanged. This also explains why the grain size of the samples annealed at 400 °C and 450 °C remains unchanged. The grain size increases as the annealing temperature is further increased (Figure 1f–j and Figure 5). During grain growth, the GBCD changes. When the temperature is increased from 600 °C to 700 °C, the grain size sharply increases from 7.2 μm to 42.4 μm.

**Figure 6:**
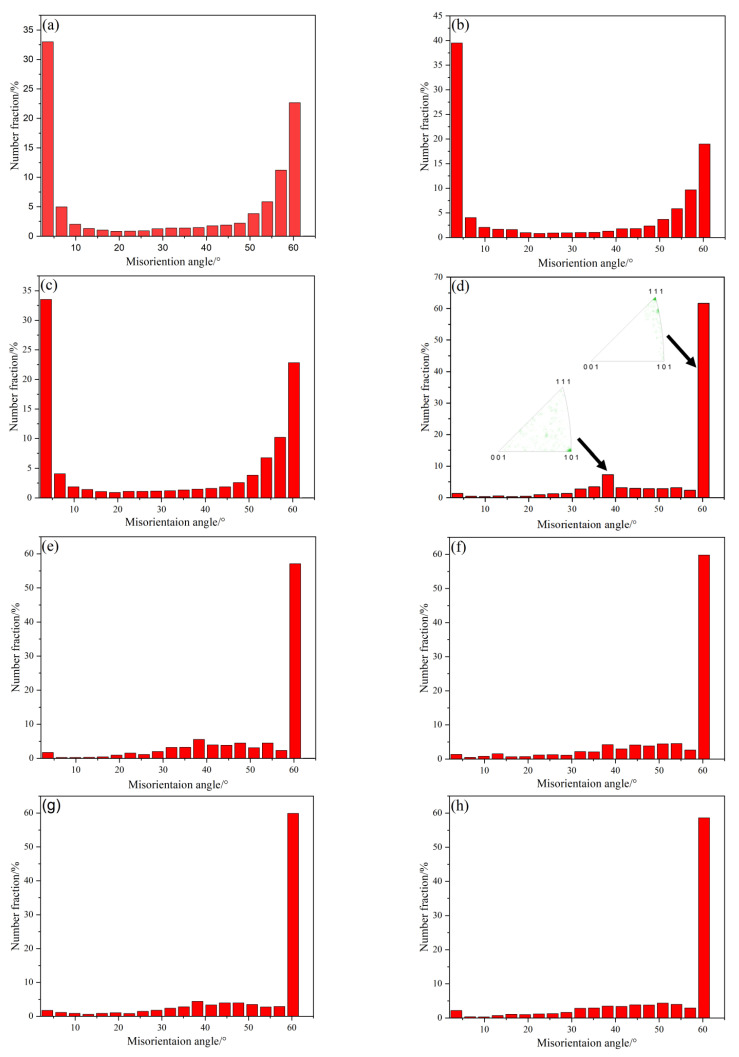

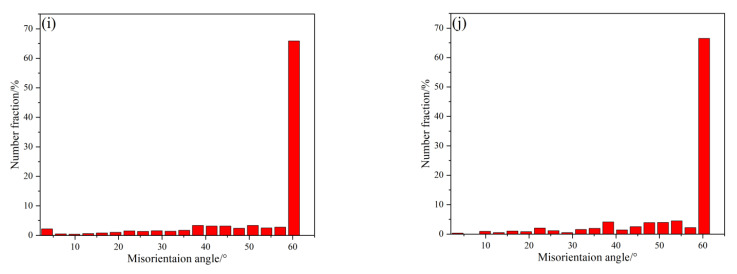
The misorientation distribution maps of the as-received sample (**a**) and annealed samples at 300 °C (**b**), 350 °C (**c**), 400 °C (**d**), 450 °C (**e**), 500 °C (**f**), 550 °C (**g**), 600 °C (**h**), 650 °C (**i**), and 700 °C (**j**) for 1 h.

Figure 6 shows the orientation difference histograms describing the GBCD of the as-received sample and the different annealed samples. For the as-received sample and the samples prepared at 300 °C and 350 °C, Figure 6a–c clearly display bimodal distributions of the misorientation with essentially the same angles. The peak with a higher intensity corresponds to the low-angle peak of LAGBs, while the peak with a lower intensity is mainly concentrated at 60° and has a rotation axis of <111>, corresponding to a twinning orientation peak. In the histogram for the sample annealed at 400 °C, no low-angle peak attributed to LAGBs is observed, indicating that the sample undergoes recrystallization while the intensity of the twinning orientation peak significantly increases. An additional small peak is present near 38.9° (Figure 6d), with a rotation axis of <110> corresponding to Σ9. The peak near 32°, which corresponds to Σ27, also has a relatively high intensity. These results indicate that during the SIBM, the GB migration can introduce low-energy SBs, such as Σ9 and Σ27, on the migrating HAGBs, accompanied by the formation of many new Σ3 boundaries. These two higher-order boundaries (Σ9 and Σ27) can potentially be obtained through the boundary decomposition model [38] or the Σ3 regeneration model [14,34]. The interaction of the two Σ3 boundaries (Σ3 + Σ3 → Σ9) produces a Σ9 boundary, which can interact with another Σ3 boundary to generate a new, movable Σ3_ic_ (Σ9 + Σ3 → Σ3), rather than Σ27 (Σ9 + Σ3 → Σ27). This is also seen in Figure 6d, where the fraction of the Σ3 boundaries is relatively high, while that of the Σ27 boundaries is relatively low. Therefore, the study results tend to support the latter mechanism. The interaction of the low-energy Σ9 and Σ27 boundaries with the incoherent Σ3_ic_ boundaries can effectively optimize the GBCD of phosphorus bronze, thereby disrupting the connectivity of the RHAGB network in phosphorus bronze [39,40]. As the temperature continues to increase, the remaining LAGBs disappear, and the peak intensity attributed to the Σ9 and Σ27 boundaries gradually decreases. The peaks related to the higher-order boundaries (Σ9 and Σ27) all decrease in intensity (Figure 6e–j). In particular, at high temperatures (700 °C), the peak intensity attributed to the Σ3 boundary remains constant (Figure 6e–h), and then increases (Figure 6i,j), while the peak intensity attributed to the Σ9 and Σ27 boundaries decreases. These results may be related to the changes in the GBCD as the grains increase in size.

**Figure 7:**
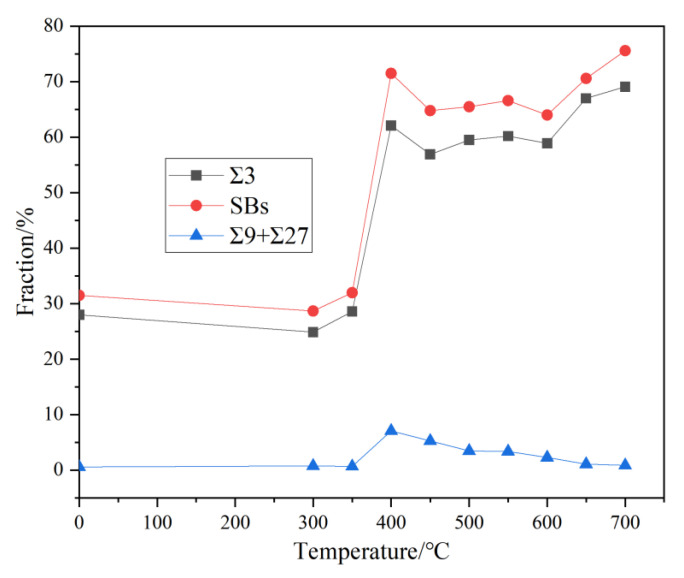
The variation in the grain boundary length fraction for the as-received sample and the samples annealed at different temperatures.

Figure 7 shows the variation in the GB length fraction for the as-received sample and the samples annealed at different temperatures. As shown in Figure 7, in the as-received sample, the length fraction of the total low ΣCSL boundaries *f_SBs_* is 31.5%, with Σ3^n^ (1 ≤ n ≤ 3) boundaries accounting for 28.6%. For a face-centered cubic material with a low SFE, this value is below the 40% threshold reported in the related literature [41]. Only when this threshold is exceeded can an optimized GBCD be obtained. Therefore, the optimization of the properties of the as-received material is difficult, and a material with the optimal GBCD required for the finished strip cannot be obtained. It is necessary to study how to maintain a consistent grain size and explore the GBCD optimization. As expected, after annealing the samples for 1 h at 300–350 °C, no significant changes are observed in the relative length fractions of different GBs. However, for the sample annealed at 400 °C, *f_SBs_* is up to 71.5%, and *f_Σ3_* and *f_Σ9+Σ27_* are 62.1% and 7.1%, respectively. A constant grain size and an excellent GBCD are observed. Furthermore, for the 450–600 °C annealed samples, the *f_SBs_* values do not continue to increase, but actually decrease. However, high values of approximately 64–66.6% are maintained, while *f_Σ9+Σ27_* gradually decreases as the temperature continues to increase. For the samples annealed at 650 °C and 700 °C, the overall *f_SBs_* values further increase similar to the *f_Σ3_* values. Meanwhile, the *f_Σ9+Σ27_* values further decrease and become almost negligible. To optimize the GBCD for phosphorus bronze, it is necessary to achieve a sufficiently high *f_Σ3_* value. The *f_Σ9+Σ27_* value is also important. Therefore, compared to those obtained with other low-SFE TMP GBE processes [42,43], the *f_SBs_* value obtained in this study is higher, indicating that the TMP of phosphorus bronze is feasible.

**Figure 8:**
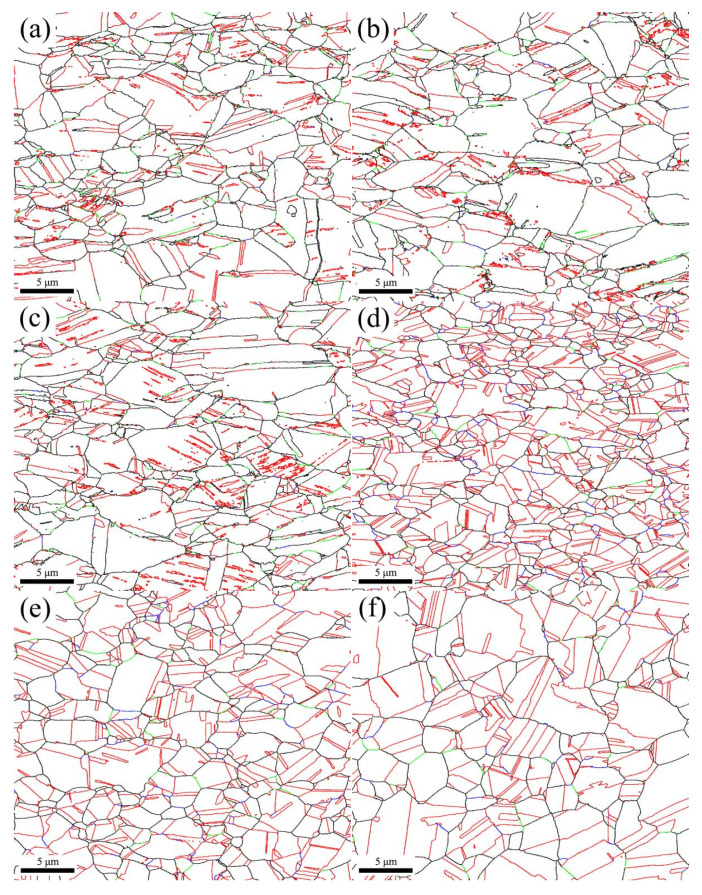

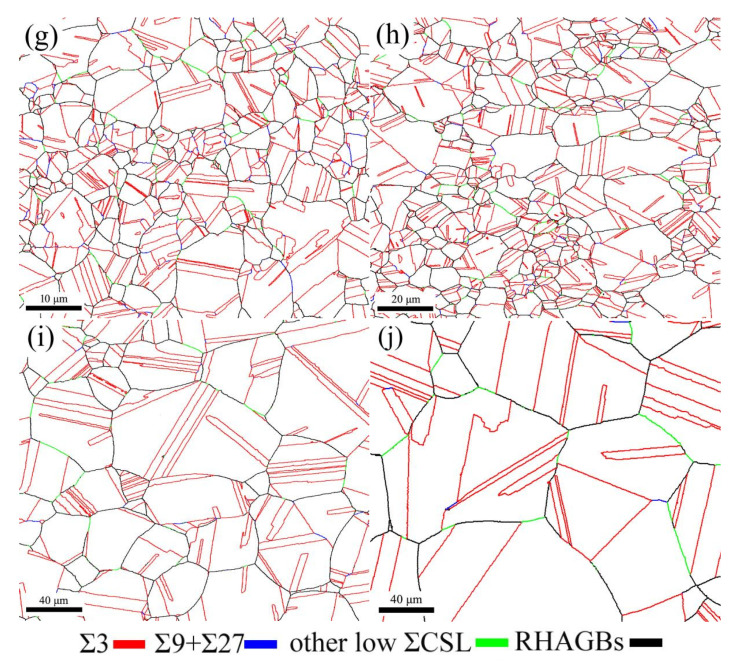
Color-coded grain boundary maps of the as-received sample (**a**) and annealed samples at 300 °C (**b**), 350 °C (**c**), 400 °C (**d**), 450 °C (**e**), 500 °C (**f**), 550 °C (**g**), 600 °C (**h**), 650 °C (**i**), and 700 °C (**j**) for 1 h (black lines represent RHAGBs, red lines represent Σ3 boundaries, blue lines represent Σ9 + Σ27 boundaries, and green lines represent other low ΣCSL boundaries).

**Figure 9:**
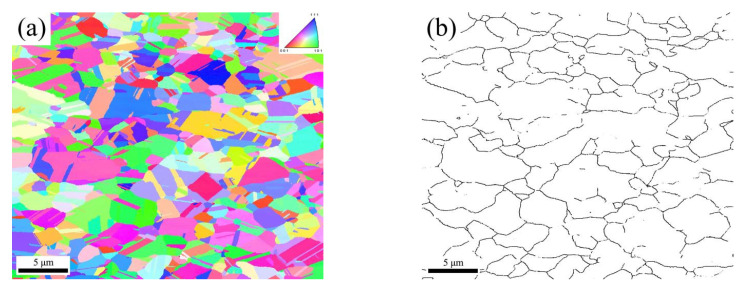
IPF map and the corresponding RHAGBs network map of the 400 °C sample. (**a**) IPF map (**b**) RHAGB network reconstruction.

The connectivity of the RHAGB network is also an important indicator for evaluating the GBCD optimization. Improved connectivity of the RHAGB network has been reported in the relevant literature [9,35]; thus, in addition to the statistical analysis of different GB fractions, in this study, the color-coded grain boundary maps of the as-received sample and the samples annealed at different temperatures are shown in Figure 8, in which the Σ3 are shown in red, the Σ9 and Σ27 in blue, the other low ΣCSL in green, and RHAGBs in black. The relatively low *f_SBs_* value of the as-received sample shown in Figure 8a indicates that its RHAGB network maintains good connectivity without disruption. For the samples annealed at 300 °C and 350 °C, with no recrystallization, the relative length fraction of the SBs remains essentially unchanged, with their values remaining at a low level, indicating that the original connectivity of the RHAGB network is maintained (Figure 8b,c). In the samples annealed at 400–600 °C, the connectivity of the RHAGB network is disrupted to some extent (Figure 8d–h). In particular, in the sample annealed at 400 °C, the grain size remains essentially unchanged, and the RHAGB network is largely disrupted, as shown in the RHAGB network map in Figure 9. For the samples annealed at 650 °C and 700 °C, although the *f_SBs_* and *f_Σ3_* values are relatively high, the low *f_Σ9+Σ27_* value indicates that the small Σ9 and Σ27 segments are unable to interact with the Σ3 boundaries, thereby failing to disrupt the RHAGB connectivity. The main reason for this finding lies in the fact that the immovable coherent twin boundaries (Σ3_c_) within the Σ3 boundaries, which mostly span the entire grain or terminate within the grain, contribute nothing to the RHAGB network adjustment [44]. Within the temperature range of 650–700 °C, the grain grows, resulting in many coherent twin boundaries (Σ3_c_) that span across the grains. On the contrary, for the incoherent twin boundaries (Σ3_ic_), because of the migration of movable incoherent twin boundaries (Σ3_ic_), new GBs are formed behind the migration front, introducing low-energy boundaries (Σ9 and Σ27) that increase the fraction of SBs. These SBs form bridges with each other, forming triple junctions, and interact with each other to disrupt the RHAGB network. This, in turn, facilitates the GBCD optimization of phosphorus bronze.

**Figure 10:**
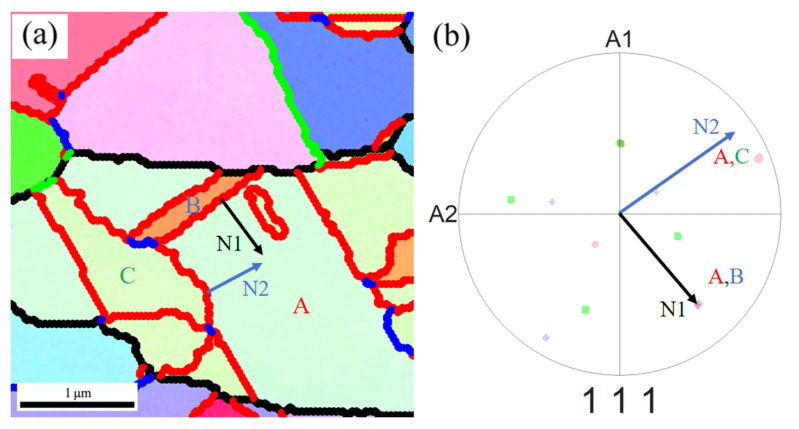
The determination of Σ3c and Σ3ic using single-section trace analysis: (**a**) IPF map; the red lines represent Σ3 boundaries and the black lines represent RHAGBs, respectively; (**b**) {111} pole figure.

The coherent twin boundaries (Σ3_c_) and the incoherent twin boundaries (Σ3_ic_) within GBs cannot be distinguished through a simple misorientation analysis. Σ3_c_ and Σ3_ic_ can be distinguished via a single-section trace analysis proposed in the literature [45,46]. In this paper, the IPF map and the {111} pole figure are extracted from a typical region in Figure 1d, as shown in Figure 10. The trace normals of the two types of Σ3 boundaries are represented by N1 (the normal to the GB between grains A and B) and N2 (the normal to the GB between grains A and C). As seen in the {111} pole figure in Figure 10b, N1 passes through the coincidence projections of grains A (red pole) and B (blue pole) well, thereby verifying that the straight GB between them is a Σ3_c_. However, for the curved GB between grains A (red pole) and C (green pole), there is a clear deviation between N2 and the coincidence projections of the two grains, indicating that it corresponds to a Σ3_ic_. In fact, the majority of Σ3 boundaries in the sample annealed at 400 °C are confirmed to be Σ3_ic_ (Figure 8d), which, according to the related literature [9,39], is closely related to the strain already present during recrystallization. Due to the high mobility of the Σ3_ic_ boundaries, they can react with the existing Σ3^n^-type GBs during migration to form a large number of Σ3^n^-type triple junctions, mainly Σ3–Σ3–Σ9 and Σ3–Σ9–Σ27 [14,16]. This ultimately effectively disrupts the RHAGB network connectivity in the sample annealed at 400 °C (Figure 8d and Figure 9b).

As previously described, with further increases in the annealing temperature, the fraction of Σ3_c_ boundaries gradually increases (Figure 8e–j), and as seen in Table 3, after the annealing temperature reaches 450 °C, the LAGBs are fully consumed. Therefore, Σ3_c_ is thought to be related to grain growth after the full consumption of LAGBs [37,47]. The formation of the Σ3_c_ boundaries is consistent with the twin growth mechanism proposed by Dash [48], Gleiter [49], etc. In addition, within the temperature range of 450–600 °C, the total fraction of the low ΣCSL boundaries decreases compared to that at 400 °C. Detwinning occurs in the recrystallized grains, but the increase in the average grain size is insignificant. When the temperature continues to increase to 650–700 °C, the average grain size significantly increases, and the total fraction of the low ΣCSL boundaries continues to increase. Therefore, as the temperature increases, the decrease in the Σ3_ic_ boundaries suppresses the formation of the Σ9 and Σ27 boundaries, ultimately leading to a decrease in the value of *f_Σ9+Σ27_*. This is also why the length fraction of the Σ9 and Σ27 boundaries gradually decreases, while that of the Σ3_c_ boundaries gradually increases. Therefore, annealing at higher temperatures does not help optimize the GBCD of phosphorus bronze mainly because the Σ3_c_ boundaries are not beneficial in disrupting the RHAGB connectivity. Moreover, for practical considerations, the grain size increases as the temperature increases. Therefore, to obtain a smaller grain, the annealing temperature of the phosphorus bronze strip should be controlled below 450 °C during annealing to ensure that the subsequent finished strip products possess excellent mechanical properties and high bending workability.

### 3.2. Effect of GBCD Optimization on Bending Properties

**Figure 11:**
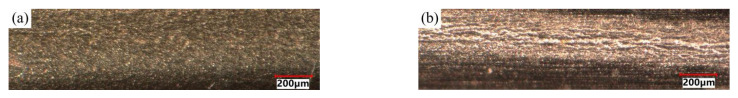
Surface morphology of 90° bending test with R/t = 0 in the BW of the GBCD-optimized fine-grained sample (**a**) and the unoptimized coarse-grained sample (**b**) with the same mechanical properties.

The fraction of boundary types, grain size, and tensile strength test results of GBCD-optimized fine-grained and unoptimized coarse-grained samples are presented in Table 4. The results presented in Table 4 reveal that the length fraction *f_SBs_* of the total low ΣCSL boundaries is 55.2%, which far exceeds the 40% threshold [41], and *f_Σ__3_* is 48 %. The optimized grain boundary characteristic distribution (GBCD) can be obtained only when the aforementioned threshold is exceeded. A 90° bending test with R/t = 0 in the BW was conducted on the GBCD-optimized fine-grained sample and the unoptimized coarse-grained sample with the same mechanical properties. The results are shown in Figure 11. Upon bending, the tensile side surface of the GBCD-optimized fine-grained sample shows no defects, such as orange peel (Figure 11a), which is consistent with Rank 1. In contrast, the tensile side surface of the unoptimized coarse-grained sample exhibits heavy orange peel and some localized areas with shallow cracks (Figure 11b), which is consistent with Rank 4. Therefore, the bending property of phosphorus bronze in the BW direction is improved as a result of fine grain and GBCD optimization. Lee et al. [50] investigated the effects of postheat treatment on the microstructure, uniaxial tensile properties, and three-point bending behavior of an extruded AZ80 alloy. The results demonstrated that grain size influences both tensile and bending properties. A strong correlation was observed between bending and tensile properties, with finer grain sizes leading to enhanced bendability [22,23]. The influence of grain boundary fraction on bending properties is also consistent with the findings reported by Oda et al. [24].

**Figure 12:**
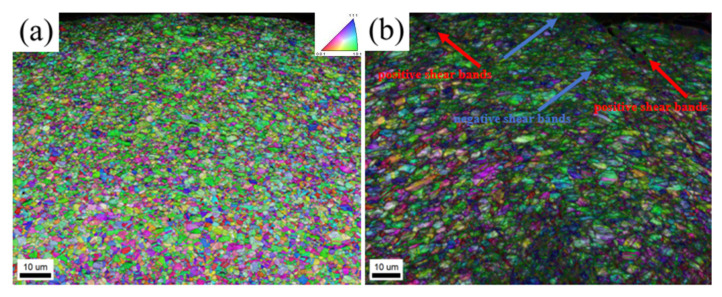
IPF + IQ maps of the cross-section of GBCD-optimized fine-grained (**a**) and unoptimized coarse-grained tensile side samples (**b**) after bending.

Further EBSD observation of the bending cross-section of tensile side (Figure 12) reveals that the GBCD-optimized fine-grained sample has no defects (Figure 12a). In contrast, the unoptimized coarse-grained sample has an uneven, stepped appearance with numerous shear bands present in the depressions, including both positive (red arrows) and negative (blue arrows) shear bands [51]. The angle of the positive shear bands is approximately 40°, while that of the negative shear bands is approximately 36°, consistent with the findings reported in the previous literature [52] showing that shear deformation tends to occur between 30° and 40°. Shear bands, as stress concentration points, are prone to inducing localized shear and crack formation. Figure 12b clearly shows many shear bands at the crack location. Therefore, the change in the surface shape at the bending location and a local shear deformation are observed.

The formation of specific shear bands and cracks can be described as follows: initially, local shear deformation occurs as a result of bending, causing the sample to exhibit surface steps, which leads to stress concentration at the steps and the initiation of a new shear deformation. With the formation of new shear bands, the stress concentration is induced at the surface grooves. Subsequently, many cracks are generated as a result of the extensive shear displacement within the shear bands. Therefore, the formation of the shear bands and the changes in the surface shape constitute a vicious cycle that promotes crack formation [52]. The EBSD analysis shows that cracks form along the shear bands, and the reason for the bending-induced cracks is the shear band.

The fine-grained phosphorus bronze obtained through GBCD optimization exhibits excellent bending properties attributed to four main reasons. First, grain refinement allows the stress during bending deformation to be distributed across a greater number of grains, thereby reducing the degree of uneven deformation and resulting in a relatively small stress concentration that can also effectively hinder crack propagation. Second, a high fraction of SBs is beneficial for enhancing the ability of dislocations to continuously multiply [11,53,54], indicating that SBs, especially Σ3 boundaries, have a high transfer ability allowing the sample with optimized GBCD to promote the reaction and proliferation of dislocations, hinder the migration of dislocations less, release the nearby stress, and improve the plasticity [55,56,57,58]. In contrast, the local dislocation accumulation at RHAGBs severely restricts the multiplication of dislocations, thereby impeding dislocation migration and complicating their passage through grain boundaries, which in turn leads to stress concentration. Third, the SBs treated with GBCD exhibit an improved uniformity of the strain distribution during bending [10], allowing the phosphorus bronze strip to exhibit good bending workability, even when subjected to a large bending deformation. In contrast, RHAGBs, due to their high interfacial energy, facilitate the formation and expansion of shear bands. Fourth, SBs have a relatively stable structure and exhibit good plastic deformation transferability that hinders the propagation of shear bands [12,59,60]. A high fraction of SBs can effectively delay the onset of deformation instability, resulting in superior bending workability.

## 4. Conclusions

In this study, the microstructural characteristics of a phosphorus bronze strip with 20% cold rolling reduction under annealing conditions of 300–700 °C were investigated by using EBSD technology. The variation patterns of its GBCD were examined, and the effect of GBCD optimization on bending properties was discussed. The main conclusions are summarized as follows:

(1) The deformed phosphorus bronze strip begins to recrystallize when the annealing temperature is increased to 400 °C. Within the range of 400–450 °C, the grain size remains essentially unchanged. The grains grow as the annealing temperature is further increased. The grains grow rapidly when the temperature exceeds 600 °C;
(2) When annealed at 400 °C, phosphorus bronze produces a large number of SBs, with the *f_SBs_* value reaching as high as 71.5% and the *f_Σ9+Σ27_* value reaching a maximum of 7.1%. The average grain size is 1.6 μm. At 700 °C, the largest *f_SBs_* value of 75.6% is observed, but the Σ9 and Σ27 boundaries are almost nonexistent. Further, the Σ3_ic_ boundaries are replaced to some extent by the Σ3_c_ boundaries, which does not promote the GBCD optimization;
(3) Compared to the unoptimized coarse-grained sample, the GBCD-optimized fine-grained sample exhibits smooth surfaces without orange peel when bent at 90° with R/t = 0 in the BW, demonstrating excellent bending workability.

## Figures and Tables

**Table 1:** Chemical composition of phosphorus bronze (wt%).

Element	Sn	P	Zn	Fe	Pb	Cu
Content	8.0	0.14	0.01	0.01	0.001	Bal.

**Table 2:** Variations in grain size and Σ3 boundary fractions (%) across five sampled regions (a–e) in 400 °C-annealed samples.

Sample	Σ3	Σ9	Σ27	Low-ΣCSL	Grain Size
a	62.1	4.7	2.4	71.5	1.60
b	61.8	4.3	2.5	71.2	1.65
c	63.2	4.8	2.5	72.8	1.55
d	62.9	4.6	2.3	71.9	1.68
e	63.4	5.0	2.5	73.1	1.53
Average	62.7	4.7	2.4	72.1	1.60
Standard deviation	0.57	0.21	0.07	0.67	0.05

**Table 3:** The fraction of low-angle grain boundaries (LAGBs) in the as-received sample and annealing samples at 300~700 °C for 1 h.

Sample	As-Received	300 °C	350 °C	400 °C	450 °C	500 °C	550 °C	600 °C	650 °C	700 °C
Fraction/%	47.5	41.4	41.1	4.5	4.1	1.2	1.2	1.1	0.9	0.8

**Table 4:** GBCD-optimized fine-grained and unoptimized coarse-grained samples boundary type ratio, grain size, and tensile strength results.

Sample	Σ3/%	Σ9 + Σ27/%	Other ΣCSL/%	Total Low ΣCSL/%	Grain Size/μm	Tensile Strength/MPa
GBCD-optimized fine-grained	48.0	2.7	0.6	55.2	1.5	621
Unoptimized coarse-grained	22.8	1.2	0.7	26.8	5.6	620

## Data Availability

The original contributions presented in this study are included in this article. Further inquiries can be directed to the corresponding authors.
